# Effect of Constant Illumination on the Morphofunctional State and Rhythmostasis of Rat Livers at Experimental Toxic Injury

**DOI:** 10.3390/ijms252212476

**Published:** 2024-11-20

**Authors:** Sevil A. Grabeklis, Maria A. Kozlova, Lyudmila M. Mikhaleva, Alexander M. Dygai, Rositsa A. Vandysheva, Anna I. Anurkina, David A. Areshidze

**Affiliations:** 1 Avtsyn Research Institute of Human Morphology of Petrovsky National Research Centre of Surgery, 117418 Moscow, Russia; 2 Research Institute of General Pathology and Pathophysiology, 125315 Moscow, Russia

**Keywords:** liver, circadian rhythm, hepatocyte, carbon tetrachloride, melatonin

## Abstract

The effect of dark deprivation on the morphofunctional state and rhythmostasis of the liver under CCl_4_ toxic exposure has been studied. The relevance of this study is due to the fact that the hepatotoxic effect of carbon tetrachloride on the liver is well studied, but there are very few data on the relationship between CCl_4_ intoxication and circadian biorhythms, and most of the studies consider the susceptibility of the organism in general and of the liver in particular to the influence of CCl_4_ in some separate periods of the rhythm, but not the influence of this chemical agent on the structure of the whole rhythm. In addition, earlier studies indicate that light disturbance causes certain changes in the morphofunctional state of the liver and the structure of the circadian rhythm of a number of parameters. As a result of this study, we found that the effect of CCl_4_ in conditions of prolonged dark deprivation causes more significant structural and functional changes in hepatocytes, as well as leading to significant changes in the circadian rhythms of a number of parameters, which was not observed in the action of CCl_4_ as a monofactor. We assume that the severity of structural and functional changes is due to the light-induced deficiency of melatonin, which has hepatoprotective properties. Thus, the mechanisms of CCl_4_ action on CRs under conditions of light regime violations leave a large number of questions requiring further study, including the role of melatonin in these processes.

## 1. Introduction

One of the most significant anthropogenic factors that has an unfavorable impact on an organism in the modern world is the violation of the light–dark regime, in particular, light pollution [1,2]. This term refers to the disturbance of the natural light regime by artificial light at night. The pollution of the night sky was first noticed by astronomers in the first half of the twentieth century [3], when it began to interfere with their stellar observations, and this phenomenon soon attracted the attention of a wider public [4]. Artificial light at night is crucial for human daily life but also pollutes the wildlife ecosystems’ environment. With an estimated average annual increase of 9.6% (due to citizen science data), light pollution is one of the most pressing drivers of current global change [5], and it has become increasingly clear that the loss of night has serious psychological, health, socioeconomic, and ecological consequences. As a modulating factor for biological systems, natural light regimes, unlike other environmental conditions such as temperature, have remained relatively constant over all the great eras of Earth’s history. This makes light pollution a great modern problem to which organisms did not have the evolutionary ability to adapt [6].

Light pollution causing desynchronosis is associated with a number of social aspects of modern life: prolonged interaction with electronic devices, modes of labor activity (overtime and shift work, the need for remote interaction with workers from other time zones), 24 h city lighting, transmeridian flights (jetlag), etc. [7]. Light regime disturbance in the form of light pollution exerts its effects in two ways: it disrupts the circadian rhythmostasis (the presence of a rhythm with a prolonged preservation of its basic parameters) of both the whole body and of its individual systems, and it suppresses the nocturnal secretion of melatonin by the pineal gland [8].

Thus, violation of the lighting regime causes at least a decrease in the adaptive capabilities of an organism and its systems, and the prolonged existence of an organism in such conditions has the consequence of accelerated aging and the development of cancer and metabolic pathologies, as well as diseases of the gastrointestinal tract (GIT) and cardiovascular system [9,10,11].

The relationship between light pollution and the violation of carbohydrate and lipid metabolism, along with the development of non-alcoholic fatty liver disease, biliary cirrhosis, malignant neoplasms of the liver, and a number of other pathologies of this principal organ, has been established [12,13,14,15].

The realization of the circadian rhythmicity of the liver is carried out by a complex regulatory system, including central regulatory links (suprachiasmatic nuclei of the hypothalamus, pineal gland), peripheral links (glands of internal secretion), and the inner cell “clock” (*Bmal1*, *Clock*, *Per*, and *Cry* families of genes) [16,17].

Thus, it was shown in experiments that carbon tetrachloride (CCl_4_) administration in the morning hours promotes a more pronounced cytotoxic and proinflammatory effect of the latter in comparison with administration in the evening hours. Data from J.V Bruckner [18] show a dependence of the severity of liver damage on the time of CCl_4_ action, but they attribute their results to the feeding of the animals. In contrast, studies by Skrzypińska-Gawrysiak M. [19] indicate that the most toxic effect of CCl_4_ is observed in the evening hours. A single study by Paulsen J.E. (1990) shows that CCl_4_ administration can alter the rhythm of DNA expression in hepatocytes [20].

Several studies have been devoted to the relationship between the effect of CCl_4_ and the work of clock genes. It was found that disruption of *Per2* and nuclear receptor *Rev-Erb-α* expression intensifies liver fibrotization. However, no data on the effect of CCl_4_ on the structure of liver circadian rhythms were found in the literature [21,22].

We considered it relevant to investigate the effect of dark deprivation on the morphofunctional state and rhythmostasis of the liver under conditions of the CCl_4_ toxic effect. This is due to the fact that the hepatotoxic effect of carbon tetrachloride on the liver is well studied, but there are very few data on the relationship between CCl_4_ intoxication and circadian biorhythms, and most of the studies consider the susceptibility of the organism as a whole and of the liver in particular to the influence of carbon tetrachloride in some separate rhythm periods, but not the influence of this substance on the structure of the whole rhythm. In addition, earlier studies indicate that light disturbance causes certain changes in the morphofunctional state of the liver and in the structure of the circadian rhythm of a number of parameters [23,24].

## 2. Results

### 2.1. Results of Morphological Study

In the rats of the control group, the morphological picture of the liver matched the age norm: the structure of the hepatic cords consisting of polygonal hepatocytes with a round nucleus located in the center of the cell was preserved. The hepatocytes of animals of this group had no any signs of functional tension or pathological changes.

In the animals of group I, the structure of the liver underwent quite pronounced changes. In the partially preserved organ, the hepatic lobules could be defined; a significant part of the hepatocytes had no signs of pathological changes, but in some hepatocytes there was small- and large-droplet fatty dystrophy. In addition, there were both separate necrotized hepatocytes and foci of necrosis. There was lymphocyte infiltration near the hepatic tracts, with lysis of erythrocytes in the capillaries.

**Figure 1:**
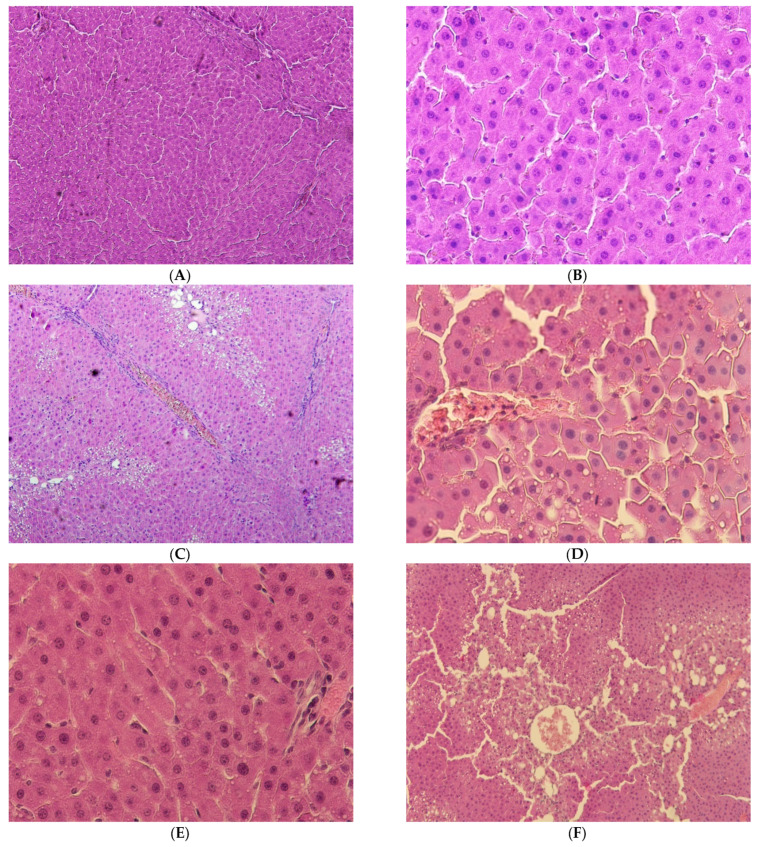

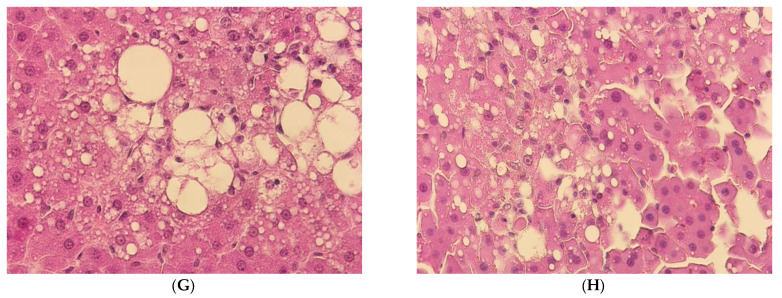
Liver of rats: (**A**) control group, hematoxylin and eosin, ×100; (**B**) control group, hematoxylin and eosin, ×400; (**C**) group I, hematoxylin and eosin, ×100; (**D**,**E**) group I, hematoxylin and eosin, ×400; (**F**) group II, hematoxylin and eosin, ×100; (**G**,**H**) group II, hematoxylin and eosin, ×400.

In the rats of the second group, destruction of hepatic lobules was observed; large-droplet fatty dystrophy in the majority of hepatocytes and significant foci of centrilobular necrosis of hepatocytes were also noted (Figure 1).

In the animals of the second group, the pathomorphological changes caused by CCl_4_ application are much more pronounced, which is manifested in a greater number of hepatocytes in the state of fatty dystrophy and necrosis and in a decreased proportion of binuclear hepatocytes both in relation to the control and to the parameters of the rats of the first group (Table 1).

The micromorphometric studies show that in animals of both groups, there is a decrease in the cross-sectional area of the hepatocyte nuclei against the background of the growth of the mean area of the cell itself and a decrease in the NCR, the indices of the second group being different from those of the first group (Table 2).

### 2.2. Results of Immunohistochemical Studies

The proportion of *Ki-67+* hepatocytes in the livers of rats of the first experimental group, amounting to 8.14 ± 0.52%, is significantly higher than that of the control group (1.04 ± 0.38%). At the same time, in the animals of the second group, the proportion of such cells practically does not differ from the proportion in the livers of control rats.

**Figure 2:**
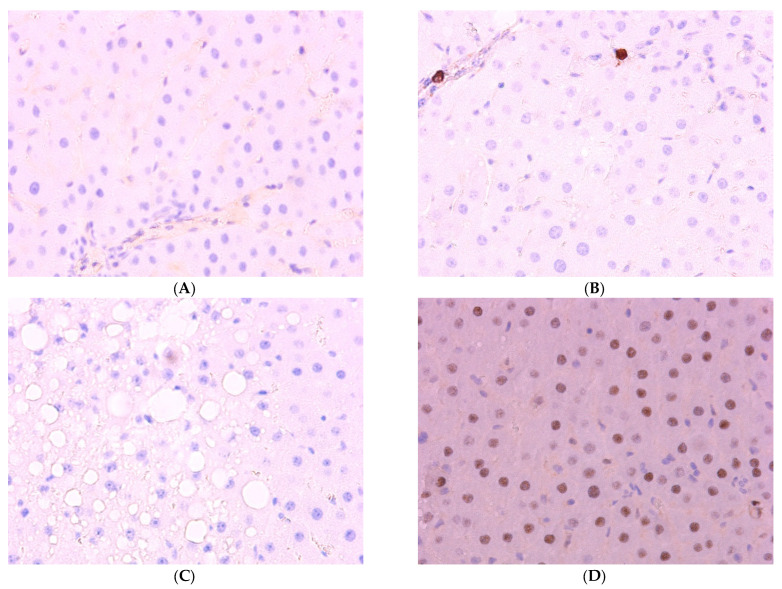

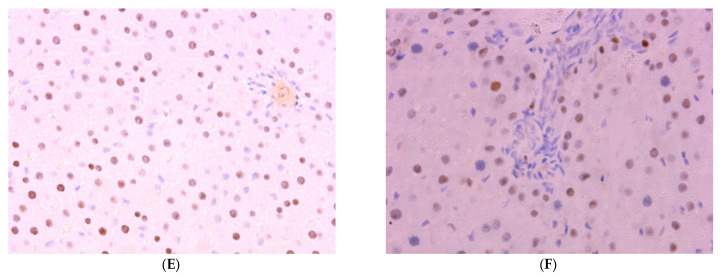
Results of ICH studies: (**A**) control group, *Ki-67*; (**B**) group I, *Ki-67*; (**C**) group II, *Ki-67*. It can be seen that single cells with reaction results are present in the field of view only in animals of the first experimental group. (**D**) Control group, *Per2*; (**E**) group I, *Per2*; (**F**) group II, *Per2*, ×400.

In turn, the proportion of hepatocytes expressing *Per2* was 47.41 ± 6.58% in the control and 43.22 ± 5.81% in the livers of animals of the first group, but it decreases to 21.57 ± 3.80% in the rats of the second group (Figure 2).

### 2.3. Results of Biochemical and Immunoassay Tests

In the course of the study, it was found that both separately and combined with constant illumination, the application of CCl_4_ leads to unidirectional changes in the studied parameters, manifested in a decrease in the content of melatonin, total protein, and albumin, but an increase in the content of glucose and in the activity of the studied aminotransferases. It is noteworthy that the indices of the second group, except for the glucose level, are different from the results obtained from the animals of the first group (Table 3).

### 2.4. Results of the Characterization of Circadian Rhythms of the Studied Parameters

The conducted study allowed us to establish that all investigated parameters are characterized by reliable circadian rhythmicity. At the same time, exposure to carbon tetrachloride under fixed lighting conditions causes destruction of the rhythms of NCR, ALT, and AST and a significant rearrangement of the rhythms of the nucleus and hepatocyte cross-sectional area, as well as of *Per2* expression. The amplitude and acrophase of the rhythms of *Ki-67*, total protein, and albumin expression change to a lesser extent, while the rhythms of melatonin and glucose content appear to be the most stable.

The action of carbon tetrachloride under conditions of constant lighting leads to the destruction of all studied CRs except the glucose rhythm (Table 4).

## 3. Discussion and Conclusions

As a result of the study, it was established that morphological and functional changes of the liver found in animals of the first group are classical for the picture of toxic liver damage by CCl_4_.

In particular, we found the development of the fatty degeneration of hepatocytes, the appearance of necrotized cells, the compensatory increase in the proportion of binuclear hepatocytes against the background of the hypertrophy of the preserved liver cells, and the increased proliferative activity in the organ, manifested in the increased expression of *Ki-67* [25,26]. Hepatocyte hypertrophy under conditions of toxic damage to the organ is caused by the retention of sodium and water ions in the cell [27].

The study of liver functions shows a consequential increase in the activity of aminotransferases due to the destruction of hepatocytes, a decrease in the levels of total protein and albumin, which reflects a decrease in the protein-synthesizing function of the liver, and an increase in glucose content which is caused by the impaired accumulation of glycogen in the liver.

The basis of these events is the biotransformation of CCl_4_, which results in the formation of toxic products that cause the hepatotoxicity of this compound. The effect of CCl_4_, which results in the formation of toxic products that cause the hepatotoxicity of this compound, proceeds in several well-studied stages. CCl_4_ penetrates into hepatocytes and forms free radicals that activate peroxidation, which leads to the occurrence of persistent liver disorders, both structural and functional [28,29]. Toxic liver damage in this case proceeds in the following sequence: reductive dehalogenation, covalent binding of radicals, inhibition of protein synthesis, fat accumulation, loss of calcium homeostasis, apoptosis, and fibrosis [30,31,32].

At the same time, we found a slight decrease in the melatonin content in the blood of rats of the first group, which is probably caused by the toxic effect of CCl_4_ on the pineal gland and is presumably associated with the abnormalities seen in the liver.

Melatonin is a powerful antioxidant [33,34,35], which also has a hepatoprotective effect. A number of studies have shown that the use of melatonin, especially at the early stages of the development of non-alcoholic liver disease in experiments, leads to the attenuation of steatosis, the reduction in aminotransferase levels, and the normalization of lipid metabolism [36,37,38]. Melatonin also prevents the development of the EPS stress often observed in hepatocytes in steatosis as well as in CCl_4_ exposure [39,40]. The hepatoprotective effects of melatonin have been observed in experimental liver cirrhosis [41,42], as well as in liver tumors [43,44]. The administration of exogenous melatonin largely offsets the fibrogenic effects of CCl_4_ by stimulating glutathionase synthesis by increasing glutamylcysteine synthase activity as well as superoxide dismutase production [45,46,47].

In animals of the second group, the differences from the control animals are similar. At the same time, pathomorphological and morphometric changes are more pronounced, differing from the parameters of the rats of the first group. The greater degree of hypertrophy of nuclei and hepatocytes in this group is due to the fact that the compensation of disturbed liver functions in these conditions can be realized by increasing the ploidy of nuclei and the formation of binuclear cells, as well as by the compensatory hypertrophy of cells [48]. In addition, hepatocyte hypertrophy may be associated with impaired cytoskeletal integrity, the maintenance of which is promoted by melatonin [49,50]. However, the less successful character of adaptation processes in the livers of rats of this group is manifested by a decrease in the proportion of binuclear hepatocytes and by an unchanged level of *Ki-67* expression relative to the control values.

The studied functional indices also differ significantly from the values of both the control animals and the rats of the first group. In addition, *Per2* expression decreases in the hepatocytes of this group.

Analyzing the results of the studies of the daily dynamics of the investigated parameters, it was established that due to the toxic effect of CCl_4_, there is a rearrangement of the CRs of morphometric parameters, including the expression of *Ki-67* and *Per2*, and the destruction of the CRs of NCR, AST, and ALT activity.

In the animals of the second group, all circadian rhythms except the rhythm of blood glucose content are destroyed.

Our earlier studies have shown that under conditions of constant illumination in rats with unchanged micromorphometric parameters, the expression of *Ki-67* and *Per2* increased, the levels of total protein and albumin decreased, and the AST activity and glucose level increased. Only melatonin and *Ki-67* expression rhythms were destroyed in the same conditions among the investigated CRs [36,38]. Thus, the significant breakdown of rhythmostasis observed in the animals of the second group is the result of the combined effect of dark deprivation and carbon tetrachloride intoxication. The reduced expression of *Ki-67* and *Per2* can be attributed to the same reasons.

To all appearances, the greater degree of liver damage in the rats of the second group was caused by a decrease in the level of melatonin, which is characterized by hepatoprotective properties. Thus, a number of studies have shown that the use of melatonin, especially in the early stages of the development of non-alcoholic liver disease in experiments, leads to the weakening of steatosis, the reduction in aminotransferase levels, and the normalization of lipid metabolism [47,48,49]. Melatonin also prevents the development of stress of the EPR, often observed in hepatocytes during steatosis, as well as during exposure to CCl_4_ [25,26]. The hepatoprotective effect of melatonin has been observed in experimental liver cirrhosis [40,41], as well as in liver tumors [39,40].

It was shown that fibrogenesis in the liver caused by the action of CCl_4_ occurs against the background of an increase in the content of MDA and NF-kB and in the production of TNF-α, IL-1, and other proinflammatory cytokines. At the same time, the activity of antioxidant enzymes decreases. A number of studies have shown that the use of exogenous melatonin significantly mitigates the fibrogenic effect of CCl_4_ by stimulating the synthesis of glutathionase by increasing the activity of glutamylcysteine synthase, as well as the production of superoxide dismutase [46,51,52].

This study allows us to say that the combined effect of dark deprivation and CCl_4_ causes a significant disruption of the chronostructure of the CRs of the studied substances. Apparently, this is associated with the disruption of the rhythm of melatonin production itself and with a decrease in its level, as well as with the decrease in *Per2* expression revealed by our study. It is clear that CCl_4_ in the background of melatonin deficiency causes disruption of the normal expression of *Per2*—the most important component of the mechanism of circadian rhythm maintenance in the cell. A single study by Paulsen J.E. (1990) [20], showing that CCl_4_ administration can change the rhythm of DNA expression in hepatocytes, speaks in favor of this fact. Some studies have shown that disruption of *Per2* and *Rev-Erba* nuclear receptor expression intensifies liver fibrotization [21,22].

Thus, we found that the effect of CCl_4_ in conditions of prolonged dark deprivation causes more significant structural and functional changes in hepatocytes, as well as significant changes in the circadian rhythms of a number of parameters. We would tend to assume that the severity of structural and functional changes is due to the light-induced deficiency of melatonin, which has hepatoprotective properties. Therefore, the mechanisms of CCl_4_ action on CRs under conditions of light regime violations leave a large number of questions requiring further study, including the role of melatonin in these processes.

## 4. Materials and Methods

### 4.1. Object of the Study

This study was conducted on 120 male rats of Wistar outbred stock (6 months old), with a body weight of 300.0 ± 35.8 g.

Animals were taken from the Research and Production Enterprise “Laboratory Animal Nursery ‘Pushchino’ of Branch of the FSBIS Shemyakin and Ovchinnikov Institute of Bioorganic Chemistry of the Russian Academy of Sciences” (IBCh RAS). All animals were kept under standard vivarium conditions, with ad libitum access to drinking water and briquetted food. Initially, all of the rats were kept under natural daylight. The keeping of the animals and the experimental implementation were made in accordance with the European Convention for the Protection of Vertebrate Animals used for Experimental and other Scientific Purposes (Strasbourg, 18 March 1986). The research was approved by the Bioethical Committee of the Avtsyn Research Institute of Human Morphology of Federal state budgetary scientific institution “Petrovsky National Research Centre of Surgery”, protocol No. 27/3 from 11 October 2021.

### 4.2. Design of Study

Rats were divided into 3 equal groups.

The control group (*n* = 40) was kept under a fixed light regime (light/dark ratio of 12:12 h with lights on at 8:00 and off at 20:00).

Group I (CCl_4_; *n* = 40) was kept under the same conditions as the controls, but the rats were injected intraperitoneally with CCl_4_ (0.2 mL/100 g b.w. in olive oil (1:1)) every three days within three weeks. Each animal received 7 injections [53].

Group II (CCl_4_ + CL; *n* = 40) was exposed to the same treatment, but while kept under a regime of constant light (CL).

Euthanasia was carried out three weeks after the start of the experiment by decapitation using a guillotine method. Animals were removed from the experiment using the time series method (regular time intervals), four times a day, at 9:00, 15:00, 21:00, and 3:00, with 10 animals sacrificed at each time point to ensure the statistical validity of the results. Previously, the rectal temperature of the animals was measured and blood sampling for hematological and biochemical studies was made. After sacrifice, evisceration was performed.

The number of animals is due to the need to ensure the reliability of the calculation of the circadian rhythms of the organism using the cosinor technique for the analysis of a time series. The number of animals in the experiment should be such as to level out the outliers determined by the individual characteristics of the animals, which can have a determining influence on the results of the experiment. According to the generalized literature data, the minimum number of animals in the experiment, necessary for the power of most statistical tests to be in 80%, and the level of their reliability to be less than 0.05, is close to 10. Thus, to obtain reliable results, our experiment used 10 animals for each time point and, accordingly, 40 animals in each group [54,55,56].

Decapitation is preferred over chemical euthanasia in studies of this type due to two reasons. First of all, chemical euthanasia methods modulate the measurements of the biochemical parameters of blood plasma and serum in comparison with the norm (for example, CO_2_ causes acidosis and potassium chloride prevents analysis of serum potassium ion levels); moreover, anesthetic agents can directly affect tissue viability or parameters. Chemical agents may directly damage tissues (for example, intraperitoneal alcohol and intraperitoneal pentobarbital both diminish the tinctorial qualities in histologic sections) [57]. In addition, in a chronobiologic study, it is important to sacrifice all animals in the shortest possible time interval during a time point for the correct accuracy of the calculation of the circadian rhythms of the studied parameters. Of all euthanasia methods, decapitation is the most consistent with this requirement.

### 4.3. Morphological, Morphometric, and Histochemical Methods

The liver samples were fixed in 10% neutral buffered formalin with further passage through alcohols of increasing concentration and xylol and poured into Histomix histological medium (BioVitrum, Moscow, Russia). Serial histological sections (5–6 μm) were made on a Leica SM2010 R rotary microtome (Leica Biosystems Nussloch GmbH, Nussloch, Germany). Hematoxylin–eosin staining was performed according to the standard technique.

Serial frozen sections of liver (6–8 μm) were prepared using a freezing table for the MFT -01 “Unicon” microtome and their standard staining with a solution of Sudan-III in 70% ethyl alcohol was performed to confirm the presence of fatty degeneration in the hepatocytes.

Microscopy of histological preparations was performed using a Leica DM 2500 digital microscope with a Leica DFC 290 digital camera (Leica Biosystems Nussloch GmbH, Nussloch, Germany)). From each sample, 10 photographs of randomly selected fields of view were taken at magnifications of ×400 and ×1000, with which karyo- and cytometry were subsequently performed [58].

With the use of the Qupath 0.5.1 software package with the appropriate plugins [30], the cross-sectional areas of nuclei (area of nucleus, S_n_) and the cross-sectional areas of cells (area of cell, S_c_) were determined. The measurements were made in micrometers after preliminary geometric calibration on an object-micrometer scale digitized with the same magnification. Micromorphometry was performed only for cells without signs of pathological changes. The proportion of binuclear hepatocytes was also determined.

The necrotic index was calculated as a % of necrotic cells from the total number of hepatocytes in the field of view.

The nuclear–cytoplasmic ratio was calculated by the following formula:

NCR = S_n_/S_c_.

Steatosis (% of hepatocytes containing lipid droplets) was scored using the non-alcoholic fatty liver disease (NAFLD) activity scoring protocol (NAS). Steatosis was scored as follows: 0, <5%; 1, 5–33%; 2, >34–65%; 3, >66% of hepatocytes containing lipid droplets [59].

### 4.4. Immunohistochemical Methods

To provide immunohistochemical (IHC) reactions, liver sections were dewaxed, rehydrated, and processed with 3% hydrogen peroxide solution to provide the blocking of endogenous peroxidase. After the unmasking of antigens by boiling in citrate buffer (pH 6.0), the slices were put into an Ultra V Block (Thermo Fisher Scientific, Waltham, MA, USA) solution. Immunohistochemical reactions with primary antibodies were carried out.

The following is a list of the used antibodies:

*Ki-67*—Rabbit polyclonal (Cloud-Clone Corp., Katy, TX, USA), 1:300);

*Per2*—Rabbit polyclonal (Cloud-Clone Corp., Katy, TX, USA), 1:200).

Sections were incubated with antibodies for 60 min at room temperature. The UltraVision Quanto Detection System (Thermo Fisher Scientific, Waltham, MA, USA) set was used as a detection system.

Reactions with the replacement of primary antibodies with phosphate buffer solution (PBS) were set out as controls.

After processing, the slides were removed, dehydrated in alcohols of ascending concentrations and xylene according to the standard scheme, and embedded in the BioMount mounting medium (BioVitrum, Moscow, Russia).

The results of the IHC reactions were assessed as the proportion of stained cell nuclei (due to localization of the antigen) in relation to the total number of hepatocytes. The evaluation was made in 4 fields of view (×400). Cells stained due to IHC reactions were counted in the preparations, and then the corresponding index was calculated as the ratio of stained cells to the total number of cells (%).

### 4.5. Biochemical Methods

The levels of total protein, albumin, alanine aminotransferase (ALT), aspartate aminotransferase (AST), and glucose were defined in the blood plasma of the rats using a StatFax-3300 analyzer (AWARENESS Technology Inc., Palm City, FL, USA) with the corresponding Spinreact kits (Spinreact, Barcelona, Spain).

### 4.6. Immunoassay Methods

Quantitative determination of the melatonin concentration in the blood was performed by enzyme immunoassay using a StatFax 4200 analyzer (AWARENESS Technology Inc., Palm City, FL, USA) with the reagent kit “ELISA Kit for Melatonin sulphate”, Cloud-Clone Corp., (CCC, Katy, TX, USA).

### 4.7. Methods for Statistical Processing

The obtained data were analyzed using the “GraphPad Prism 6.0” software by calculating the mean values, standard deviation, and mean error of the arithmetic mean. The data in the text, tables, and graphs are presented as Mean ± SD. Numerical rows characterizing the daily fluctuations of the physiological rhythms of the studied parameters were subjected to mathematical processing, on the basis of which the group chronograms were drawn. The shapes of the chronograms were analyzed and the average daily values were determined. To identify differences between control and experimental groups, univariate analyses (with the use of ANOVA or Kruskal–Wallis where appropriate) for clinical parameters and laboratories were made. Differences were considered statistically significant at *p* < 0.05.

For determination of the amplitude and acrophase of the circadian rhythms of the studied parameters, we performed cosinor-based analysis using the CosinorEllipse2006-1.1 program [24,60].

Intensity of steatosis (% of hepatocytes with lipid droplets in their cytoplasm) was calculated by means of the non-alcoholic fatty liver disease (NAFLD) activity scoring protocol (NAS), and the results were as follows: 0 points, <5%; 1 point, 5–33%; 2 points, >34–65%; 3 points, >66% of hepatocytes containing lipid droplets. Necrosis was determined as follows: 0 = none, 1 = 1–5%, 2 = 6–20%, 3 ≥ 20% [61].

## Figures and Tables

**Table 1:** Influence of CCl_4_ on some pathomorphological parameters of the livers of rats.

Group of Animals	NAS Index	Proportion of Hepatocytes Containing Lipid Droplets, %	Necrosis Index	Proportion of Necrotic Hepatocytes, %	Proportion of Binuclear Hepatocytes, %
Control group (*n* = 40)	0 ± 0	1.70 ± 0.01	0 ± 0	0.5 ± 0.04	7.04 ± 1.98
Group I (*n* = 40)	1.89 ± 0.04	33.41 ± 3.22 ***	1.0 ± 0.2 ***	4.87 ± 0.57 ***	9.97 ± 1.60 *
Group II (*n* = 40)	2.11 ± 0.07 ** ■	57.6 ± 6.90 *** ■■	2.1 ± 0.31 *** ■■	35.82 ± 4.66 *** ■■■	5.14 ± 1.44 * ■

Note: * (*p* ≤ 0.05); ** (*p* ≤ 0.005); *** (*p* ≤ 0.0005)—in comparison with the parameters of control group animals. ■ (*p* ≤ 0.05); ■■ (*p* ≤ 0.005); ■■■ (*p* ≤ 0.0005)—in comparison with the parameters of experimental group I.

**Table 2:** Micromorphometric parameters of the hepatocytes of rats.

Group of Animals	Cross-Sectional Area of Nuclei of Hepatocyte, µm^2^	Cross-Sectional Area of Hepatocyte, µm^2^	NCR
Control group, *n* = 40)	41.72 ± 2.24	186.50 ± 28.51	0.22 ± 0.05
Group I (CCl_4_, *n* = 40)	38.1 ± 3.25 *	201.10 ± 8.50 *	0.19 ± 0.04 *
Group II (CCl_4_ + CC), *n* = 40)	35.81 ± 3.05 *** ■	215.20 ± 17.18 * ■	0.16 ± 0.03 *** ■

Note: ■ (*p* ≤ 0.05); * (*p* ≤ 0.05); *** (*p* ≤ 0.0005)—in comparison with the parameters of control group animals.

**Table 3:** Biochemical parameters of the blood plasma of rats.

Group of Animals	Total Protein, g/L	Albumin, g/L	ALT, U/L	AST, U/L	Glucose, mmol/L	Melatonin, pg/mL
Control group, (*n* = 40)	69.71 ± 8.14	41.26 ± 6.54	68.35 ± 7.22	123.21 ± 14.51	7.82 ± 1.15	18.54 ± 1.21
Group I (CCl_4_, *n* = 40)	50.11 ± 5.10 ***	30.14 ± 5.22 *	87.41 ± 8.22 **	156.81 ± 18.52 *	8.91 ± 1.16 *	14.22 ± 0.91 *
Group II (CCl_4_ + CL), *n* = 40)	41.29 ± 6.1 *** ■	20.15 ± 3.25 ** ■	121.11 ± 13.55 *** ■	183.71 ± 22.1 ** ■	8.22 ± 1.72 *	4.51 ± 0.39 *** ■■■

Note: * (*p* ≤ 0.05); ** (*p* ≤ 0.005); *** (*p* ≤ 0.0005)—in comparison with the parameters of control group animals. ■ (*p* ≤ 0.05); ■■■ (*p* ≤ 0.0005)—in comparison with the parameters of experimental group I.

**Table 4:** Effect of CCl_4_ on circadian rhythms of the studied parameters.

Group of Animals	Cross-Sectional Area of Nuclei			Cross-Sectional Area of Hepatocytes		NCR	
	Acrophase	Amplitude		Acrophase	Amplitude	Acrophase	Amplitude
Control *n* = 40)	12:18	9.45		10:26	18.14	11:04	0.06
Group I (CCl_4_, n = 40)	20:24	5.14		23:44	16.88	No reliable CR	
Group II (CCl_4_ + CL), *n* = 40)	No reliable CR			No reliable CR		No reliable CR	
	*Ki-67*			*Per2*		Glucose	
	Acrophase	Amplitude		Acrophase	Amplitude	Acrophase	Amplitude
Control, *n* = 40)	6:48	0.17		3:48	7.54	13:14	1.52
Group I (CCl_4_, *n* = 40)	10:55	0.54		14:33	5.88	15:49	2.14
Group II (CCl_4_ + CL), *n* = 40)	No reliable CR			No reliable CR		14.39	5.11
	AST			ALT		Total protein	
	Acrophase	Amplitude		Acrophase	Amplitude	Acrophase	Amplitude
Control, *n* = 40)	10:48	18.56		19:08	2.03	15:26	7.25
Group I (CCl_4_, *n* = 40)	No reliable CR			No reliable CR		16:14	6.89
Group II (CCl_4_ + CL), *n* = 40)	No reliable CR			No reliable CR		No reliable CR	
	Albumin				Melatonin		
	Acrophase		Amplitude		Acrophase	Amplitude	
Control, *n* = 40)	14:39		8.66		1:27	14.18	
Group I (CCl_4_, n = 40)	11:06		9.24		2:09	16.22	
Group II (CCl_4_ + CL), *n* = 40)	No reliable CR				No reliable CR		

## Data Availability

The data presented in this study are available within the article text, tables, and figures.
